# Cognitive Dysfunction in the Early Stages of Multiple Sclerosis—How Much and How Important?

**DOI:** 10.1007/s11910-020-01045-3

**Published:** 2020-05-22

**Authors:** Magdalena Oset, Mariusz Stasiolek, Mariola Matysiak

**Affiliations:** Department of Neurology, Medical University of Lodz, Kopcinskiego 22, 90-153 Lodz, Poland

**Keywords:** Early stage of multiple sclerosis, Cognition, Cognitive dysfunction, Prognostic markers in early MS, Biomarkers in early MS

## Abstract

**Purpose of Review:**

Multiple sclerosis (MS) is a demyelinating disease of the central nervous system that mainly affects young adults and that is one of the leading causes of disability in this age group, with cognitive impairment occurring early in the course of the disease. This article summarizes the current knowledge about cognitive dysfunction in the early phase of MS, including biomarkers, MRI correlates, and its value as a prognostic marker.

**Recent Findings:**

New sets of neuropsychological tests have been established to screen for cognitive dysfunction more easily and accurately. Moreover, structural changes detected by brain MRI and several biomarkers found in cerebrospinal fluid and blood serum have been recently correlated with decreased cognitive performance. Additionally, factors influencing cognition in MS, such as disease-modifying therapy, mood disorders, and lifestyle, are better described.

**Summary:**

Cognitive impairment early in the course of MS is suggested as a prognostic factor for disease progression. However, clear-cut definitions of the early stage of MS as well as unified criteria for the diagnosis of cognitive impairment are still lacking. New and more reliable tools for evaluating cognition in MS patients should be developed and introduced into everyday practice to facilitate the implementation of effective disease-modifying therapy, cognitive rehabilitation, and lifestyle management.

## Introduction

Multiple sclerosis (MS) is a chronic inflammatory demyelinating disease of the central nervous system (CNS). MS affects mainly young adults (20–40 years old) and is one of the leading causes of neurological impairment in this age group [1••]. Apart from physical disabilities, the disease is known to cause cognitive dysfunction in up to 70% of patients throughout their lifetime [2]. However, the influence of the early phase of MS on cognition is not clearly established. According to various reports, cognitive impairment may occur in 20 to 45% of cases [3] and may even precede other symptoms by more than a year [4].

An accurate description and diagnosis of cognitive impairment in the early stage of MS are of great importance, as it may be a useful determinant of the effectiveness of potential preventive measures or a predictor of future disease progression [5]. Reducing the number of affected patients may help prevent a high unemployment rate [6•, 7] and improve the quality of life [8] for people in this group.

The high variation in the reported prevalence of cognitive deficits in early MS may be due to several reasons. First, difficulties arise in defining the early stage of MS, as it is described differently in various studies [9–15]. Most frequently, the early phase of the disease is understood as the short period after a clinically isolated syndrome (CIS) [10, 12]. However, in other studies, early MS is defined as the duration of the disease up to five years after diagnosis, or alternatively characterized as the disease phase limited by Expanded Disability Status Scale (EDSS) score below 3–3.5 [13, 14, 16]. Moreover, the definitions and the diagnostic criteria of cognitive impairment differ between studies as a result of a vast variety of applied neuropsychological tests and a lack of unified cut-off scores used over the years [15].

Cognitive impairment related to MS is usually described as heterogeneous in affected domains. It is assumed that cognitive dysfunction in MS is similar to that in subcortical dementias [3]. The most affected domains are attention, information processing speed, memory, executive functions, and visuospatial skills [2]. However, in the initial phases of MS, processing speed and executive functions are impaired, followed by dysfunctions in memory [15] or attention [2, 17]. Cortical domains such as praxis and gnosis are usually spared even in the later stages of the disease [18].

## Assessment of Cognitive Function in Multiple Sclerosis

Tests commonly used to screen for cognitive deficits in dementias, such as the Mini-Mental State Examination (MMSE) or Montreal Cognitive Assessment (MoCA), which mostly assess cortical function, are not sensitive or specific enough to test cognition in MS because other domains are typically affected in this condition [18].

One of the first batteries of neuropsychological tests presented to evaluate MS-related deficits was the Neuropsychological Screening Battery for MS (NSBMS), developed by neuroscientists from the Cognitive Function Study Group of the USA. This battery includes the Selective Reminding Test (SRT), the 7/24 Spatial Recall Test (SPART), the Paced Auditory Serial Addition Test (PASAT), and the Word List Generation Test (WLGT). Later, the same group proposed the applicability of the Brief Repeatable Battery of Neuropsychological Tests (BRB-N), supplemented with the Symbol Digit Modalities Test (SDMT) using the 10/36 SPART instead of the 7/24 version [19].

After several years, due to the need for improved diagnostic precision, a new reliable test battery named the Minimal Assessment of Cognitive Functioning in Multiple Sclerosis (MACFIMS) emerged. In this assessment, the 10/36 SPART was replaced with the Brief Visuospatial Memory Test-Revised (BVMT-R), and SRT was replaced with the California Verbal Learning Test-Second Edition (CVLT-II). Moreover, two newly developed tests were added: the Judgment of Line Orientation and the Delis-Kaplan Executive Function System, which assess executive and visuospatial functions. The BRB-N and MACFIMS performed similarly and suitably in the recognition of cognitive decline in MS [19]. Despite a high sensitivity, implementation of these batteries in clinical practice required time and money, as they were relatively long and required a trained neuropsychologist to administer. Thus, a more cost-effective way to assess cognition in MS is still in demand.

Currently, the Brief International Cognitive Assessment for Multiple Sclerosis (BICAMS) is becoming increasingly popular, especially because it can be easily implemented by clinicians and takes 15 min to complete. The BICAMS includes the SDMT, CVLT-II, and BVMT-R and is currently regarded as a recommended and widely validated screening tool for cognitive impairment in MS [20].

A diagnosis of cognitive impairment is established when a patient’s performance in at least two tests from a battery is below the normal range, considered as either 2 SD [10] or 1.5 SD below the control mean [15]. Alternatively, results below the 5th percentile of those in the control group may be taken into account as a more restrictive cut-off score [15]. According to observations suggesting that information processing speed and attention may be impaired early in MS, the SDMT (measuring these modalities) seems to be the most effective single tool to assess cognition even in the initial stages of the disease [21].

The SDMT can be administered in five min and does not require the involvement of trained neuropsychologists. Written and oral versions exist, so even patients with a physical disability are able to complete the tasks. Moreover, the SDMT is relatively free from the effects of practice [22]. Therefore, this test is considered to be particularly suitable for the detection of cognitive decline in the course of MS [23], and some researchers claim that the SDMT alone could be used as an effective measure to screen for cognitive impairment in MS [24]. Furthermore, the results of the SDMT were shown to be predictive of future cognitive decline [25] and an unemployment status [7]. Cognitive domains measured by tests included in neuropsychological batteries used in MS are summarized in Table 1.

**Table 1:** Cognitive domains measured by tests included in neuropsychological batteries used in MS

Test	Domain	Battery
Selective Reminding Tests	Verbal learning, memory	NSBMS, BRB-N
Spatial Recall Test (SPART)	Visuospatial memory	NSBMS (SPART7/24), BRB-N (SPART10/36)
Auditory Serial Addition Test	Information processing speed, working memory, divided attention	NSBMS, BRB-N, MACFIMS
Word List Generation Test	Verbal fluency	NSBMS, BRB-N, MACFIMS
Symbol Digit Modalities Test	Information processing speed, attention	BRB-N, MACFIMS, BICAMS
Controlled Oral Word Association Test	Verbal fluency	BRB-N, MACFIMS
Brief Visuospatial Memory Test-Revised	Visuospatial learning, memory	MACFIMS, BICAMS
California Verbal Learning Test-Second Edition	Verbal learning, memory	MACFIMS, BICAMS
Judgment of Line Orientation	Visuospatial perception	MACFIMS
Delis-Kaplan Executive Function System Sorting Test	Executive functions	MACFIMS

*NSBMS*, The Neuropsychological Screening Battery for MS; *BRB-N*, The Brief Repeatable Battery of Neuropsychological Tests; *MACFIMS*, The Minimal Assessment of Cognitive Functioning in Multiple Sclerosis; *BICAMS*, The Brief International Cognitive Assessment for Multiple Sclerosis

## Cognitive Dysfunction and the Clinical Course of MS

The severity of cognitive deficits differs among particular clinical courses of MS. It is assumed that cognitive dysfunction is already present in the early stage of the disease, even in patients with CIS, and progresses in a parallel manner to accumulating disability [26]. The available data indicate that cognitive decline is more prominent in progressive forms of the disease [27]. In one of the studies, an isolated decrease in phonemic fluency was observed in RRMS patients with a disease duration below three years. In the group of patients with a disease duration above 10 years, the digit span test and SDMT results indicated the patients were impaired, whereas patients with a progressive disease scored below normal in all neuropsychological tests except for the inhibition task. Interestingly, no significant differences were observed between the SP and PPMS forms [28]. In a large sample study encompassing 1040 MS patients, the prevalence of cognitive impairment in the whole group was 46.3%. This proportion differed substantially between particular phases and courses of the disease: 34.5% of patients with CIS, 44.5% of patients with RRMS, 79.4% of patients with SPMS, and 91.3% of patients with PPMS; the information processing speed was the most commonly affected domain in all the groups. Significant differences were observed between relapsing and progressive forms of the disease but not between CIS and RRMS or SPMS and PPMS patients [26]. A systematic review and meta-analysis performed on the data from 47 studies (encompassing 4460 patients) showed that PPMS patients exhibited moderately higher impairment in all cognitive domains than RRMS patients, with the differences in deficits in verbal learning, processing speed, and verbal memory being more prominent than in other domains [27]. Although differences between RRMS and SPMS may be explained with great probability by an obviously longer duration of the disease that associated with a higher lesion load and a more severe disability, similarities in SPMS and PPMS may reflect the role of neurodegeneration rate and the intensity of brain atrophy [27].

## Factors Influencing Cognitive Function in Multiple Sclerosis

Several factors are known to influence the level of cognitive dysfunction. Regular physical exercise, a lack of addictions, a healthy diet, and the proper control of comorbidities can positively affect cognition in patients with MS [29]. Growing evidence also indicates that proper disease-modifying therapy, implemented early in the course of RRMS, may stabilize or even improve cognition [30]. It was shown that treatment with interferon beta (IFNb) may improve cognition, especially in women [31]. In the COGIMUS study, cognitive decline was reduced by 32% in patients who had been treated with 44 μg of IFNb 3×/week over 3 years. Another disease-modifying therapy (DMT) shown to positively affect cognition was natalizumab. Several studies demonstrated that treatment with natalizumab exerted a positive effect on cognition, depression, and fatigue in MS patients [32–34]. Importantly, drugs used traditionally for enhancing cognition, such as acetylcholinesterase inhibitors or memantine, were indicated to be ineffective or even have negative outcomes in MS patients with cognitive problems [35].

The cognitive reserve (CR), defined as an individual’s cognitive processing ability and estimated based on the person’s years of education, IQ, and involvement in leisure activities, may explain individual differences in the cognitive status of patients with similar MRI and clinical findings [36]. A higher CR was found to be protective over the initial state of RRMS for almost all cognitive domains; however, this effect may decrease with the progression of brain damage [37, 38]. Nonetheless, the exact meaning of the CR evokes considerable controversy. Some studies showed that the influence of a CR extended over several years of the MS disease course [39], whereas others did not prove its significance in predicting the level of cognitive decline [40].

Psychiatric disorders are also considered to potentially influence cognitive abilities in MS patients. The most frequent, depression, affects approximately 30% of MS patients [41]. Several studies revealed a negative impact of depressive symptoms on cognitive function, especially on information processing speed, executive function, attention, motor function, and memory [42], but they may affect virtually all cognitive domains [43]. Another study showed that in early MS, depression accounts for a slowed information processing speed and impaired working memory but not for executive functioning [44]. Anxiety disorder, which affects approximately 22% of MS patients [41], may influence mainly episodic memory and executive function [45]. However, since depressive symptoms frequently overlap with anxiety, it is difficult to clearly distinguish between the effects of those particular disorders. In the study designed to determine the separate impact of depression and anxiety on cognition, higher depressive symptoms were associated with worse processing speed as well as lower employment, higher fatigue, and a greater physical disability, but anxiety did not show such a correlation. These findings may suggest that depression is more disabling than anxiety in MS patients and should be managed with particular care [46].

Apart from mood disorders, up to 80% of patients with MS may suffer from fatigue during their lifetime [47]. Fatigue is frequently defined as a general tiredness or lassitude even without any physical effort [35]. One of its important components that occurs in up to 90% of MS patients is cognitive fatigue, defined as a decrease in task performance as a result of continuous cognitive effort. Interestingly, cognitive fatigue was recently suggested to be a more sensitive marker of cognitive decline than measures of overall cognitive performance in the early phase of MS [48].

The effect of vitamin D supplementation on cognition has also been discussed in the context of cognitive performance. It may be beneficial, especially to healthy individuals with low serum vitamin D levels or with lower cognitive functioning. If supplementing in high doses, vitamin D was shown to improve nonverbal memory [49]. This effect was also observed in people with MS, with an indication that high levels of 25(OH)D were more effective in augmenting long-term memory [50].

Additionally, there are reports showing that smoking may have a negative impact on cognition in MS. It was also found that heavy smokers had increased cognitive impairment in comparison to nonsmokers [51]. Moreover, cognitive dysfunction was significantly more severe in patients with a longer history of addiction than in patients with a shorter history [52].

## Brain Imaging Techniques Relevant to the Assessment of Cognition in MS

Cognitive dysfunction may result from damage to various structures and connections in the CNS; therefore, many different techniques have been employed to seek appropriate imaging correlates. Magnetic resonance imaging (MRI) allows the detection of Gd-enhancing lesions, T2 lesions, T1 lesions (so-called “black holes”), and atrophy. All of those are considered to place a burden on cognition [53].

Several studies evaluated associations between the T1 and T2 lesion load and cognitive deficits. In a study of 62 patients with CIS, the authors demonstrated that deterioration in the overall cognitive score and executive function over seven years of observation could be correlated with the number of T1 lesions in the first year after CIS. Moreover, an increased number of T2 lesions in the first three months after CIS offered predictive information for the patient’s future executive function performance [54]. Additionally, it was shown that a higher T2 lesion load obtained in a short period after a CIS was connected to cognitive decline after five years [55]. Another important factor taken into consideration was early inflammatory activity, counted as the number of Gd-enhancing lesions. It was suggested that this parameter may predict memory, executive, and overall scores on neuropsychological tests after seven years of follow-up [54]. Another study showed that cognitive decline during the first 10 years of RRMS was highly associated with the occurrence of new brain T2 lesions in the frontal, temporal, and parietal lobes [56]. Interestingly, in one of the analyses, new T2 lesions were better correlated than cortical lesions with cognitive deficits, leading to the conclusion that white matter focal pathology may be more important for cognitive deficits than cortical lesions (CLs) or cortical atrophy [57]. Additionally, in RRMS, worse results for mental processing speed tests were correlated with an increased T2 lesion load, as well as with a larger normalized ventricular volume and lower normalized volumes of the whole brain, whole gray matter, and cortical gray matter. However, in this study, no significant correlation was found with white matter atrophy [58].

On the other hand, several authors described a correlation between the number of cortical lesions and cognitive impairment. For instance, in one of the studies, gray matter pathology was associated with poorer cognitive outcomes in MS, and the number of CLs correlated positively with the level of decline in the semantic word fluency (Regensburger Word Fluency Test) and working memory (PASAT) [59]. It should also be noted that different mechanisms and/or different brain imaging characteristics may be crucial for evaluating cognitive decline in particular stages of MS. In the study by Eijlers and colleagues, it was demonstrated through a longitudinal logistic regression analysis that during a five year follow-up period, cognitive decline in early RRMS was predicted by white matter integrity, whereas in late RRMS and progressive MS, cognitive deficits were predicted by cortical atrophy only [1••].

In contrast to the aforementioned results, in another study (which included 53 patients with RRMS with a disease duration of one to five years and EDSS scores ≤ 5.0), the overall cognitive impairment did not correlate with any of the demographic, clinical, or MRI variables at baseline. After two years of observation, only a decrease in brain parenchymal volume was correlated with changes in cognitive performance [60], indicating the importance of the dynamics of volumetric brain parameters.

Cortical thickness represents another anatomical and imaging parameter of potential value in the context of assessing cognitive function. It is very stable, highly heritable, and is not affected by pseudoatrophy but rather by neurodegenerative processes such as demyelination and neuronal, axonal, and synaptic loss. However, the association of cortical thickness with cognitive performance again evokes some controversy. It was demonstrated that cortical thickness was related to clinical symptoms of MS, such as physical disability, cognitive deficits, depression, and fatigue [61, 62]. A positive correlation was also found between the results of verbal memory tests (the sum score of the first five trials of the CVLT-II) and the cortical surface area in the lateral occipital, fusiform, and inferior temporal regions of the left hemisphere. However, better visuospatial memory was associated with a larger cortical volume in the supramarginal and superior temporal regions of the right hemisphere [63].

Atrophy of specific brain regions may also be of high importance in the context of cognitive function. A reduction in thalamus volume was already shown to be associated with cognitive impairment in RRMS patients with a disease duration below three years and EDSS scores below 3.0 [64] and was suggested as one of the most important predictors of cognitive performance in MS [65]. Another significant measure is the corpus callosum index (CCI). The CCI strongly correlated with corpus callosum volume and other volumetric brain features, including white matter volume and lesion volume, and it was considered to be a valid surrogate marker of brain atrophy in MS. In RRMS patients, CCI was demonstrated to correlate with PASAT and MSFC scores [66], and SDMT and Verbal Fluency Test results, but not with tests of verbal memory [67]. Additionally, the hippocampus, a brain structure crucial for memory processes, was demonstrated to be affected in the early stages of MS. Microstructural damage measured by diffusion tensor imaging (DTI) was already observed in patients with CIS, and the MRI findings were strongly correlated with long-term recall [68]. Moreover, in patients examined two to six months after CIS, performance on learning trials was correlated with hippocampal volume [69]. The magnetization transfer ratio (MTR) is believed to be an indicator of structural tissue integrity and to correlate with myelin and axonal density in the CNS. Whole brain MTR at an early stage of MS was shown to be significantly lower in the subgroup of patients with cognitive deficits compared to individuals without cognitive symptoms. This finding may suggest the existence of diffuse brain tissue changes in patients in the early phases of MS [70]. In another study assessing multiple MRI parameters, only the lower mean cortical MTR was associated with a decrease in mental processing speed in CIS patients. In the RRMS group of the same study, which only differed demographically from the CIS group by a younger age at disease onset, a normalized cortex volume was shown to be the strongest predictor of mental processing speed, followed by the CNS lesion load. These findings may suggest that structural cortical changes are the prevailing morphological correlate of the initial cognitive deficits seen in the early stages of MS [58].

Other MRI techniques and parameters have also been implicated as correlates of cognitive function in MS, including diffuse axonal loss in white matter that appears normal [54, 71]. Recently, retinal thickness measured by OCT was found to be potentially useful in clinically monitoring axonal loss in MS. In particular, one parameter, the peripapillary retinal nerve fiber layer (RNFL), was associated with brain atrophy [72]. The RNFL was shown to correlate positively with the result of the SDMT in the early stages of MS [73]. This observation seems to be of great interest considering the potential application of OCT as a noninvasive, less expensive technique that may be useful in detecting axonal loss in MS.

## Multiple Sclerosis Biomarkers Related to Cognitive Dysfunction

Several biomarkers measured in cerebrospinal fluid (CSF) or in serum have been implicated as potentially effective in monitoring the MS course from the earliest stages of the disease [74].

Recently, neurofilaments have become biomarkers of the highest interest in this area. Neurofilaments are axonal cytoskeletal proteins composed of three chains: light, medium, and heavy. They are released into body fluids when axonal damage occurs; thus, increased levels of neurofilaments have been observed in the CSF and blood serum in all stages of MS. To date, most neurofilament light and heavy chains (NfL and NfH, respectively) have been investigated in MS [75]. Increased NfL levels in the CSF were observed in patients with Gd-enhancing lesions in the brain and/or spinal cord and were independently associated with the occurrence of clinical relapses [76]. Moreover, higher levels of NfL were found in CIS patients with their condition progressing toward MS [77]. NfH levels in CSF have been correlated with brain volume reductions and disability progression over time [78]. There is also a strong positive correlation between CSF and serum NfL levels in MS patients, which indicates that serum measurements of this biomarker are potentially useful in everyday clinical practice after sufficient further validation [76]. In a preliminary fMRI study involving 21 untreated, cognitively preserved patients with CIS, higher CSF NfL levels were associated with lower activity in the putamen, while performing a task requiring increasing levels of attentional control processing. This result may suggest NfL as a marker of abnormal cognitive pathway recruitment even preceding the first clinical signs of cognitive impairment in patients in the earliest stages of MS [79]. Another study reported that higher CSF NfL levels are associated with lower scores on a word list generation assessment [80•].

The other widely investigated biomarkers are chitinase-like proteins (CHILPs). The biological role of these proteins is still elusive, but it is believed that they are involved in tissue remodeling, inflammation, and cell survival. Moreover, higher CHILP levels in CSF may reflect a high degree of axonal damage [81]. Chitinase 3-like 1 protein (CHI3L1) is a molecule suggested to play a role in inflammation and the manner in which tissue responds to injury [82]. It was found that its level in CSF predicts progression in early MS, illustrating neuronal damage from the beginning of the disease [81]. CHI3L1 was also shown to be associated with lower Trail Making Test A scores in recently diagnosed RRMS patients [80•]. Another member of this protein family, chitinase-3-like 2 protein (CHI3L2), the closest homolog of CHI3L1, was suggested as a useful biomarker of the transition from optic neuritis to MS [82]. Moreover, the increased level of CHI3L2 in patients diagnosed with optic neuritis as a first demyelinating episode was associated with poorer performance in PASAT after a 14-year follow-up period [82].

## Cognitive Impairment in Early MS as a Prognostic Factor

The reports on the progression of cognitive impairment in the course of MS are contradictory, with both the preservation and gradual decline of cognitive function being under discussion. The features of progressive cognitive deterioration were shown in a study of 30 patients who were followed over approximately six years in which a significant decline in divided attention capabilities and information processing speed was observed, whereas other domains remained stable. Notably, in this study, 83% of the patients started treatment with disease-modifying drugs during follow-up, which could significantly affect the results [83]. Another study reported that overall cognitive deterioration was evident after 10 years of observation, with an increase in the percentage of affected patients from 26 to 56% [84]. A more recent study including 24 patients with CIS who were highly suggestive of MS reported that the percentage of individuals with cognitive impairment increased from 29 to 54% after five years of follow-up [55]. On the other hand, another study presented that over five years of observation, 28% of 234 MS patients (181 with RRMS, 33 SPMS, and 20 PPMS) deteriorated in cognitive function, while the remaining 72% were cognitively stable [1••].

Several studies considered the possibility that early cognitive impairment in MS can serve as a predictor of future disease progression. It was shown that cognitive deficits at the time of CIS onset were associated with progression to MS, and the risk increased with the number of neuropsychological tests that the patient failed [12, 55]. Another study encompassing 56 patients reported that 64% of patients who failed over two tests and 88% of those who failed three tests were diagnosed with MS within a period of 3.5 ± 2.3 years, with a calculated hazard ratio of 4.0 [12]. Moreover, in another study, cognitive impairment at diagnosis was shown to possess a predictive value for the clinical progression of disabilities and cortical atrophy in MS patients after eight years of observation [85••]. In another report, a higher SDMT score was associated with a reduced likelihood of reaching 4.0 points on the EDSS over 10 years [5]. Deterioration in EDSS scores over seven years was also significantly predicted by cognitive impairment diagnosed early in the course of RRMS [86].

## Conclusion

Cognitive deficits affect more than half of MS patients and are among the most disabling. Cognitive performance is a potential predictive marker of progression of the disease and a patient’s future employment status and quality of life. Identifying cognitive impairment at the earliest stages should be a crucial part of the assessment of the patient’s clinical status. Consequently, when diagnosed at an early stage, cognitive dysfunction may suggest implementing highly effective DMTs and focusing on cognitive rehabilitation in addition to promoting a healthy lifestyle. Unfortunately, clear-cut definitions of the early stage of MS as well as unified criteria for the diagnosis of cognitive impairment are still lacking. Therefore, cognition in MS should be further investigated and extensively characterized. In parallel, new effective cognition assessment methods in MS patients should be continuously developed to become an inseparable part of the comprehensive examination of patients with a diagnosis of CIS and in further stages of the disease.
